# Information-Theoretic Causal Feature Selection via Markov Blanket Discovery for Stock Return Direction Prediction: Evidence from China

**DOI:** 10.3390/e28080847

**Published:** 2026-07-29

**Authors:** Jiamei Zhou, Hongxu Wu, Shaoze Li

**Affiliations:** 1 College of Computer and Cyber Security, Fujian Normal University, Fuzhou 350117, China; 121142024010@student.fjnu.edu.cn; 2 School of Finance and Accounting, Fujian Business University, Fuzhou 350012, China; 3 School of Economics and Management, University of Chinese Academy of Sciences, Beijing 100190, China; shaoze_li@163.com

**Keywords:** causal feature selection, Markov Blanket, conditional mutual information, IPCMB-CART, stock direction prediction, Chinese stock market

## Abstract

Stock markets are complex adaptive systems whose feature dependencies evolve over time, and identifying which signals genuinely drive returns is fundamentally a problem of information. Traditional feature selection methods and linear models rely on statistical correlations and overlook causality, which weakens their predictive performance in non-linear, evolving markets. This paper develops an information-theoretic approach to causal feature selection based on Markov Blanket discovery. We apply the Iterative Parent–Child-based search of Markov Blanket (IPCMB), whose conditional-independence tests are conditional mutual information measures, to recover the Markov Blanket of next-month returns—the minimal feature set that carries all Shannon information about the target. We then pair it with a Classification and Regression Tree (CART), whose impurity-based splitting admits an information-gain interpretation, to predict the direction of Chinese A-share returns non-linearly. Using data on 2760 stocks, IPCMB selects 13 causal features from 72 candidates, and CART forecasts whether the next month’s return is positive. The empirical results show an accuracy of 58.0%, 7.7 percentage points above the all-features CART benchmark, and a 13-month cumulative return 35.88% higher than that of the CSI 300 index. The findings indicate that selecting features according to their causal information content and combining them with interpretable tree-based prediction can support more reliable investment decisions in emerging markets.

## 1. Introduction

As data volumes grow and data become more accessible, the use of technologies such as machine learning for stock prediction and investment decision-making has attracted considerable interest from both the financial industry and academia [1]. The stock market is a rapidly evolving, complex, non-linear dynamical system [2], while big data are characterized by volume, variety, velocity, and value [3]. Traditional statistical-arbitrage and factor-investing models are challenged by their stationarity assumptions and limited ability to adapt to non-linear dynamics. Forecasting the direction of next-period returns is of first-order importance for portfolio allocation and risk management, yet a harder question lies behind it: among the hundreds of firm-level, trading, and macroeconomic signals now available, which ones actually drive returns rather than merely move with them? Because the answer depends on separating genuinely informative signals from incidental ones, stock-return prediction is fundamentally a problem of information—the perspective adopted in this paper.

On the one hand, high-dimensional data give investors access to an expanding array of factors that may influence risk premia. Traditional asset-pricing models, such as the Fama–French multifactor models, often lack the high-dimensional perspective required to capture the intricate price dynamics of financial instruments [4,5]. On the other hand, the declining density of useful information makes it difficult to exploit financial big data under the stationarity and linearity assumptions underlying models such as the Autoregressive Moving Average (ARMA) model. In addition, the rapid growth of new media has made it more difficult for researchers to identify effective predictors in complex and diverse information environments.

Two shortcomings of the standard toolkit stand out in this setting. First, conventional feature selection and linear time-series models rank predictors by statistical association—a correlation, marginal mutual information, or regression weight—and therefore cannot distinguish features that genuinely drive returns from those that are only incidentally correlated with them. In a high-dimensional and non-linear market, such associations are easily spurious or redundant: selecting features on this basis introduces noise, double-counts features that carry the same information, and overlooks signals that become informative only in combination. Consequently, predictive power tends to erode precisely when the feature space is richest.

The second shortcoming concerns interpretability. Non-linear machine-learning and deep-learning models capture market complexity much better than linear baselines, but the most accurate models, particularly deep neural networks, often operate as black boxes: a forecast can be obtained without revealing which features produced it or why. This opacity is not merely cosmetic. It limits the ability to trace a prediction to economically meaningful drivers, trust the model when the market regime shifts, and justify model-based decisions to investors or regulators. This tension is particularly acute in an emerging market such as China’s A-share market, where retail investors dominate and abrupt policy shocks move prices; both the underlying causal structure and its interpretation therefore have direct implications for real-world decisions. A method that selects features carrying genuine predictive information and uses them transparently for forecasting would address both shortcomings simultaneously.

Against this backdrop, this paper proposes the IPCMB-CART model to address the failure of traditional feature selection algorithms to account for causal relationships and the difficulty that traditional statistical models face in handling non-linear time series [6,7]. The model is based on an information-theoretic view of causal discovery. The Iterative Parent–Child-based search of Markov Blanket (IPCMB) [8] recovers the target’s Markov Blanket through conditional-independence tests that are, at their core, measures of conditional mutual information [9]. The Markov Blanket is the minimal feature set that retains all the Shannon information needed to predict the target. The selected features are then passed to a Classification and Regression Tree (CART), whose impurity-based splitting can itself be interpreted as an information-gain criterion.

The empirical study uses data on 2760 stocks in China’s A-share market and considers 72 features covering company growth, profitability, solvency, valuation, stock trading, and macroeconomic factors. We focus on monthly prediction because this horizon provides a practical alignment with firm fundamentals disclosed quarterly, semiannually, or annually and is standard in empirical asset-pricing research [4,5]. Nevertheless, IPCMB-CART is not inherently horizon-specific and can be applied to daily or weekly prediction when appropriately timed high-frequency predictors are available. The full feature set follows [10]; the complete list of the 72 candidate features is reported in Appendix A for reproducibility. Comparisons with traditional feature selection algorithms based on the Pearson Correlation Coefficient (PCC), Mutual Information (MI), and the F-test demonstrate the model’s generalization ability and robustness, in line with recent hybrid feature-selection pipelines for stock trend prediction [11].

The contribution of this paper is twofold. The first is methodological. To the best of our knowledge, this is the first study to bring Markov-Blanket-based causal feature selection into the monthly stock-return prediction pipeline, and it does so within a single information-theoretic framework that meets the three challenges above at once. Causality is addressed by IPCMB, which keeps a feature only when it carries conditional mutual information about the target beyond what the other features already supply, rather than because it is correlated with them. Interpretability follows from CART, whose tree exposes the decision path and grants each causal feature an information-gain-based importance that reads directly as its contribution to the forecast. Non-linearity is handled by the same tree, which partitions the causal feature set without imposing a linear form.

The second contribution is empirical. On a large panel of Chinese A-shares we benchmark the model against correlation-, mutual-information-, and *F*-test-based selectors, against eleven alternative Markov-Blanket learners, and against a range of competing classifiers, and we pair the classification results with a simulated trading backtest. The evidence is systematic and consistent: features chosen for their causal information content predict direction more accurately and trade more profitably than correlation-based features, which shows concretely that causal, rather than merely correlational, information can improve investment decisions in an emerging market.

The remainder of the paper is organized as follows. Section 2 reviews the literature on stock prediction. Section 3 introduces the theoretical foundations and methods underlying the IPCMB-CART model. Section 4 applies the model to the Chinese A-share market. Section 5 compares the model with alternative algorithms and methods. Finally, Section 6 summarizes the paper and outlines directions for future research.

## 2. Literature Review

Advances in feature selection and machine learning have opened new avenues for stock prediction. Researchers have applied various traditional feature selection algorithms, including the filter method of Principal Components Analysis (PCA) [12], the wrapper method of Genetic Algorithm (GA) [13], and the embedded method of Support Vector Machine (SVM) [14]. Some studies have also begun to examine causality-based feature selection in stock prediction. For instance, Deng et al. [15] generated a causal graph from financial news and incorporated the resulting causal information into a deep neural network for interpretable stock-trend prediction, reporting performance gains over state-of-the-art baselines. Nam and Seong [16] used transfer entropy and multiple-kernel learning to identify causal features linking a target company to associated companies. Chen et al. [17] proposed a multiple-cause discovery method combined with structure learning (McDSL) to eliminate spurious causal relationships. More broadly, in the econophysics literature, transfer entropy—a model-free measure of directed information flow between two series that is, in effect, a conditional mutual information, the same quantity that drives our feature-selection step—has been widely used to assess the direction and strength of information flow across financial markets. Its bias-corrected version, effective transfer entropy, has been used to trace information flow between exchange rates and stock markets [18], and such information-flow measures remain common in recent studies of financial networks and capital flows [19]. Our aim, however, is different: rather than measuring information flow between a pair of series, we use the Markov Blanket to select the smallest set of features that is conditionally informative about the target.

However, these approaches are not grounded in the causal-inference theory of Bayesian Networks (BNs) and Markov Boundaries (MBs). Causal-inference theory establishes the MB as the smallest optimal feature set that preserves the target’s original distribution [20]; in other words, the target’s MB contains sufficient information to explain it fully. In information-theoretic terms, conditioning on the MB reduces the mutual information between the target and every remaining feature to zero, making the MB a minimal sufficient statistic for predicting the target [9]. Accordingly, MB discovery algorithms can be used as a filter-based method known as causal feature selection (CFS) (In the stock-prediction literature, CFS often denotes correlation-based feature selection, which differs from the causal feature selection considered in this paper). Representative methods include constraint-based CFS algorithms such as GSMB, IAMB [21], PCMB [22], BAMB [23], and KIAMB [22], as well as score-based variants such as DMB and BSS-MB; Yu et al. [24] provide a comprehensive comparison. Applications of CFS in finance nevertheless remain scarce.

For stock-market direction prediction, researchers have explored non-linear machine-learning methods to address the curse of dimensionality. Ayyildiz and Iskenderoglu [25] compared seven machine-learning algorithms across major G7 stock indices and reported that non-linear methods such as Artificial Neural Networks (ANNs) and Support Vector Machines (SVMs) outperform linear baselines such as Logistic Regression (LR) in forecasting stock direction. Recent studies have extended deep-learning architectures in this direction: Chaudhari and Thakkar [26] employed a Backpropagation Neural Network (BPNN) and Long Short-Term Memory (LSTM), while Cui et al. [27] proposed a hypergraph tri-attention network (HGTAN). Hybrid frameworks have also attracted attention. Nabipour et al. [28] benchmarked deep-learning models such as LSTM against tree-based ensembles for stock-market prediction, while Dong and Liang [29] proposed a hybrid CNN-LSTM-GNN network for Chinese A-share prediction that combines an entropy-and-correlation feature-selection filter with graph-based temporal learning. Such filters, however, still rank features according to marginal information or correlation, whereas the present approach selects them according to their conditional contribution given the other features. Despite these advances, neural networks remain largely black boxes, limiting the analysis of how specific features drive forecasts and thereby reducing their value for investment decision-making. This paper therefore introduces the IPCMB-CART model, which integrates the CFS algorithm (IPCMB) with the non-linear prediction model (CART) to address causality, interpretability, and high-dimensionality within a single pipeline.

## 3. The Proposed Model

### 3.1. IPCMB-CART Model

**Figure 1:**
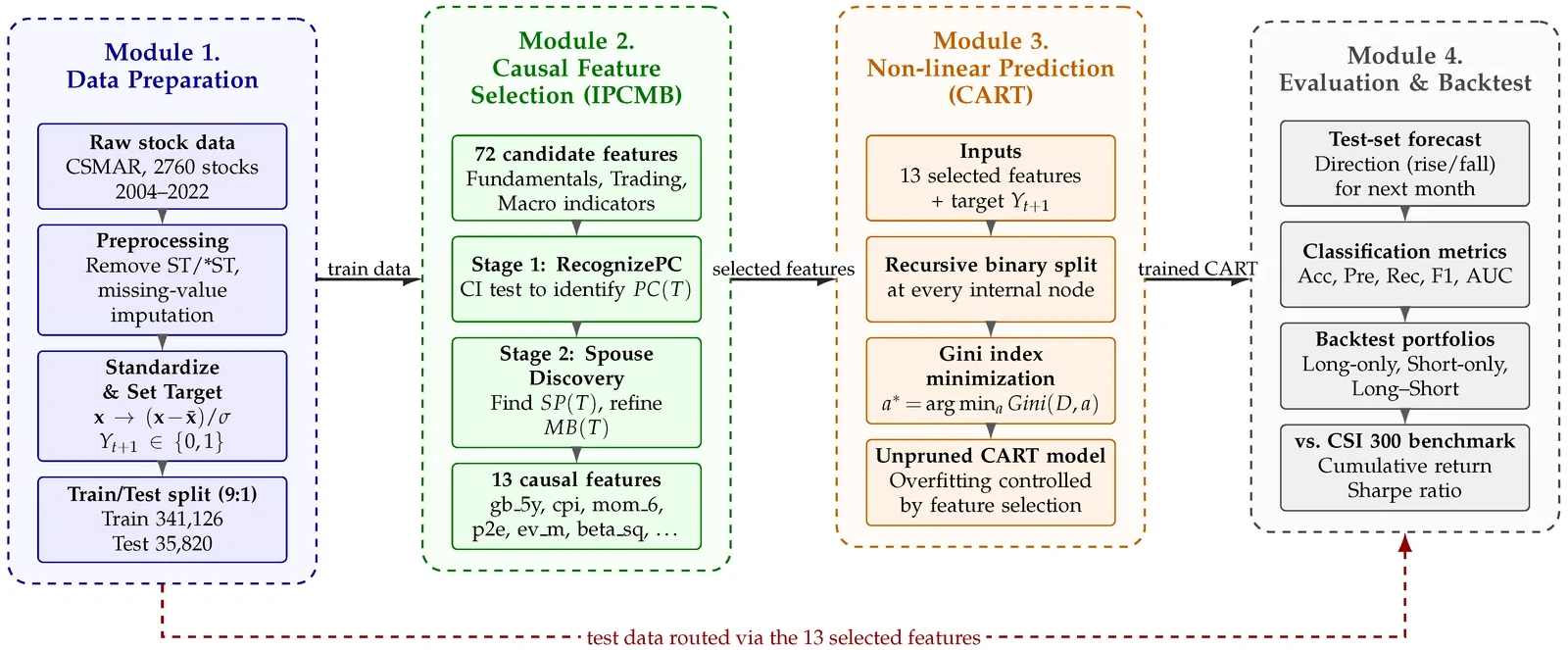
Framework of the IPCMB-CART model. Solid arrows trace the training pipeline that learns the Markov Blanket and fits the CART classifier; the dashed arrow shows the test-set path that reuses the selected features and the trained model for out-of-sample direction forecasting and backtesting.

Drawing on IPCMB’s ability to discover causal features and CART’s ability to classify non-linear data, the proposed IPCMB-CART model predicts whether each stock’s return will be positive or negative in the following month. As illustrated in Figure 1, the framework comprises four sequential modules: (i) data preparation, (ii) causal feature selection via IPCMB, (iii) non-linear prediction via CART, and (iv) evaluation and trading backtesting. The IPCMB algorithm and the CART model are described in Section 3.2 and Section 3.3, respectively.

Following the four-module framework in Figure 1, the IPCMB-CART model is implemented in four steps as follows.

Step (1) Data preparation: After data cleaning and missing-value imputation, the raw sample obtained from a database such as CSMAR is divided into training and test sets at a 9:1 ratio. Each feature in the training set is standardized to have a mean of 0 and a standard deviation of 1. The target variable is then converted into a binary indicator equal to 1 for a positive next-month stock return and 0 otherwise, with the timing shifted so that current-month features predict next-month direction.

Step (2) Causal feature selection via IPCMB: The IPCMB algorithm searches the candidate feature pool for the Markov Blanket of the target variable, which becomes the selected feature subset. The search proceeds locally rather than by learning the entire Bayesian network: IPCMB first locates the variables that are conditionally adjacent to the target (its direct causes and direct effects), then runs the same local search around each adjacent variable to pick up the spouses (other direct causes of the target’s effects), and finally merges the two groups into a single Markov Blanket.

Step (3) Non-linear prediction via CART: CART constructs the tree by evaluating candidate split points for the selected features and choosing those that minimize the Gini index. Recursive splitting continues until each leaf is pure or no further valid split remains. Overfitting is controlled upstream through IPCMB feature selection rather than by pruning the tree: discarding noisy and redundant predictors and retaining only causal features curbs the fitting of spurious patterns.

Step (4) Evaluation and trading backtest: The test set is standardized using parameters carried over from the training set, and the selected features are fed into the trained CART, which outputs a binary forecast of next-month direction. The forecasts are evaluated by classification accuracy and related metrics, and the binary signals are translated into a simulated trading strategy whose cumulative returns are compared with the CSI 300 benchmark.

The IPCMB-CART model is essentially a classification model that differs from conventional models in its feature selection step. Traditional classifiers typically rely on wrapper-based feature selection [6,24] to remove redundant and ineffective features during training, but this approach has several limitations in stock-prediction settings. Wrapper algorithms are tied to a specific learner, so the selected feature subset may not transfer well across model families or to autocorrelated and evolving stock features. They evaluate features mostly by their individual contribution to a target metric and often miss higher-order interactions such as causal dependencies. Because feature selection is performed jointly with model fitting, they tend to over-optimize on accuracy and overfit, which is especially harmful for time-series data such as stock returns. The proposed model addresses these issues by replacing the wrapper step with the IPCMB algorithm, which selects causal features as a model-agnostic preprocessing stage.

### 3.2. IPCMB

The Iterative Parent–Child-based search of MB (IPCMB) [8] is an extension of the PCMB family that recovers the Markov Blanket through a breadth-first local search. Because the search is local, IPCMB does not need to estimate the target variable’s entire Bayesian network, which keeps the search space small and makes the algorithm tractable for the high-dimensional, sample-rich datasets used in stock prediction. This feature makes IPCMB particularly suitable for the monthly-return setting: estimating a complete BN for stock returns is computationally and statistically demanding, and this difficulty has motivated alternative approaches to causal discovery, such as transfer entropy [16].

At the heart of IPCMB is a conditional-independence (CI) test. For the target *T*, a candidate feature *X*, and a conditioning set *S*, the statistic $I_{D}\left(X,T|S\right)$ used in Algorithm 1 is the conditional mutual information between *X* and *T* given *S*, estimated on the data *D*:

(1) $$I\left(X;T|S\right)=\sum_{s}\sum_{x}\sum_{t}p\left(x,t,s\right)\;\log\frac{p(x,t|s)}{p(x|s)\;p(t|s)}=H\left(T|S\right)-H\left(T|X,S\right),$$

where H(·∣·) denotes conditional Shannon entropy. Conditional mutual information is non-negative and equals zero if and only if *X* and *T* are conditionally independent given *S* [9]; the threshold ε in Algorithm 1 therefore tests whether *X* carries any additional information about *T* once *S* is known. In other words, IPCMB retains a feature only when it reduces the target’s uncertainty (its conditional entropy) beyond what the rest of the conditioning set already explains.

In practice this conditional mutual information is estimated from finite samples. Because every feature is first rescaled to zero mean and unit variance (Section 4.1, Equation (9)), we implement the CI test through Fisher’s *z*-transform of the partial correlation $\rho_{X,T|S}$, which for jointly Gaussian variables is a monotone function of the conditional mutual information:

(2) $$I\left(X;T|S\right)=-\frac{1}{2}\ln\;\left(1-\rho_{X,T|S}^{2}\right).$$

Testing $\rho_{X,T|S}=0$ at a fixed significance level is thus equivalent to thresholding I(X;T∣S) at the corresponding ε in Algorithm 1, which keeps the search computationally light on the high-dimensional panel. The Gaussian link in Equation (2) is only an approximation. In our panel about three-quarters of the candidate features are clearly skewed and most have heavy tails, and standardization changes their scale but not this shape.

We nonetheless rely on the approximation for two reasons. The test enters the algorithm only through the ranking of partial correlations against a common threshold, so an informative feature tends to stay above the threshold even when its distribution is not exactly normal. The blanket it produces also holds up out of sample, outperforming the correlation- and *F*-test-based subsets in Section 5 (Table 7), which would be hard to explain if the test were badly miscalibrated. As a simple check, replacing the ordinary correlation with a rank-based (Spearman) one leaves the sign of each selected feature’s association with the target unchanged for nearly all of them, so the selection does not appear to hinge on normality. A fully nonparametric test, such as a kernel or nearest-neighbor estimator of conditional mutual information or a copula-based test, would relax the assumption further and is a natural way to extend the method.
**Algorithm 1** Pseudocode for the IPCMB algorithm [8].**Process 1: RecognizePC****Input:** Dataset *D*; target variable *T*; adjacency set ADJ(T); threshold ε1:**function**RecognizePC(D,T,ADJ(T),ε)2:    NonPC←⌀3:    cutSetSize←04:    **repeat**5:        **for all** X∈ADJ(T) **do**6:           **for all** S⊆ADJ(T)∖{X} such that |S|=cutSetSize **do**7:               **if** $I_{D}\left(X,T|S\right)\leq \varepsilon$ **then**8:                   NonPC←NonPC∪{X}9:                   $Sepset_{T,X}\leftarrow S$10:                   **break**11:               **end if**12:           **end for**13:        **end for**14:        ADJ(T)←ADJ(T)∖NonPC15:        NonPC←⌀16:        cutSetSize←cutSetSize+117:    **until** cutSetSize≥|ADJ(T)|18:    **return** ADJ(T)                ▹CanPC(T)19:**end function****Process 2: IPCMB****Input:** Dataset *D* over variable set *U*; target variable *T*; threshold ε20:**function**IPCMB(D,T,U,ε)21:    CanADJ(T)←U∖{T}22:    CanPC(T)← RecognizePC(D,T,CanADJ(T),ε)23:    MB(T)←CanPC(T)24:    **for all** X∈CanPC(T) **do**25:        CanADJ(X)←U∖{X}26:        CanPC(X)← RecognizePC(D,X,CanADJ(X),ε)27:        **if** T∉CanPC(X) **then**28:           MB(T)←MB(T)∖{X}29:           **continue**30:        **end if**31:        **for all** $Y\in CanPC\left(X\right)\setminus \left(\{T\}\cup MB(T)\right)$ **do**32:           **if** $I_{D}\;\left(T,Y|Sepset_{T,Y}\cup \left\{X\right\}\right)>\varepsilon$ **then**33:               MB(T)←MB(T)∪{Y}34:           **end if**35:        **end for**36:    **end for**37:    **return** MB(T)38:**end function**

The Markov Blanket inherits a direct information-theoretic meaning. Writing *U* for the full feature set, MB(T) is the minimal subset such that

(3) $$I\left(T;\;U\setminus (MB(T)\cup \{T\})\;|\;MB(T)\right)=0,$$

that is, once the blanket is known the target shares no further mutual information with any other feature. MB(T) is thus the minimal sufficient feature set for *T* in the Shannon sense, which is exactly the property that makes it the theoretically optimal input for a downstream predictor and motivates its use as a causal feature selector.

**Figure 2:**
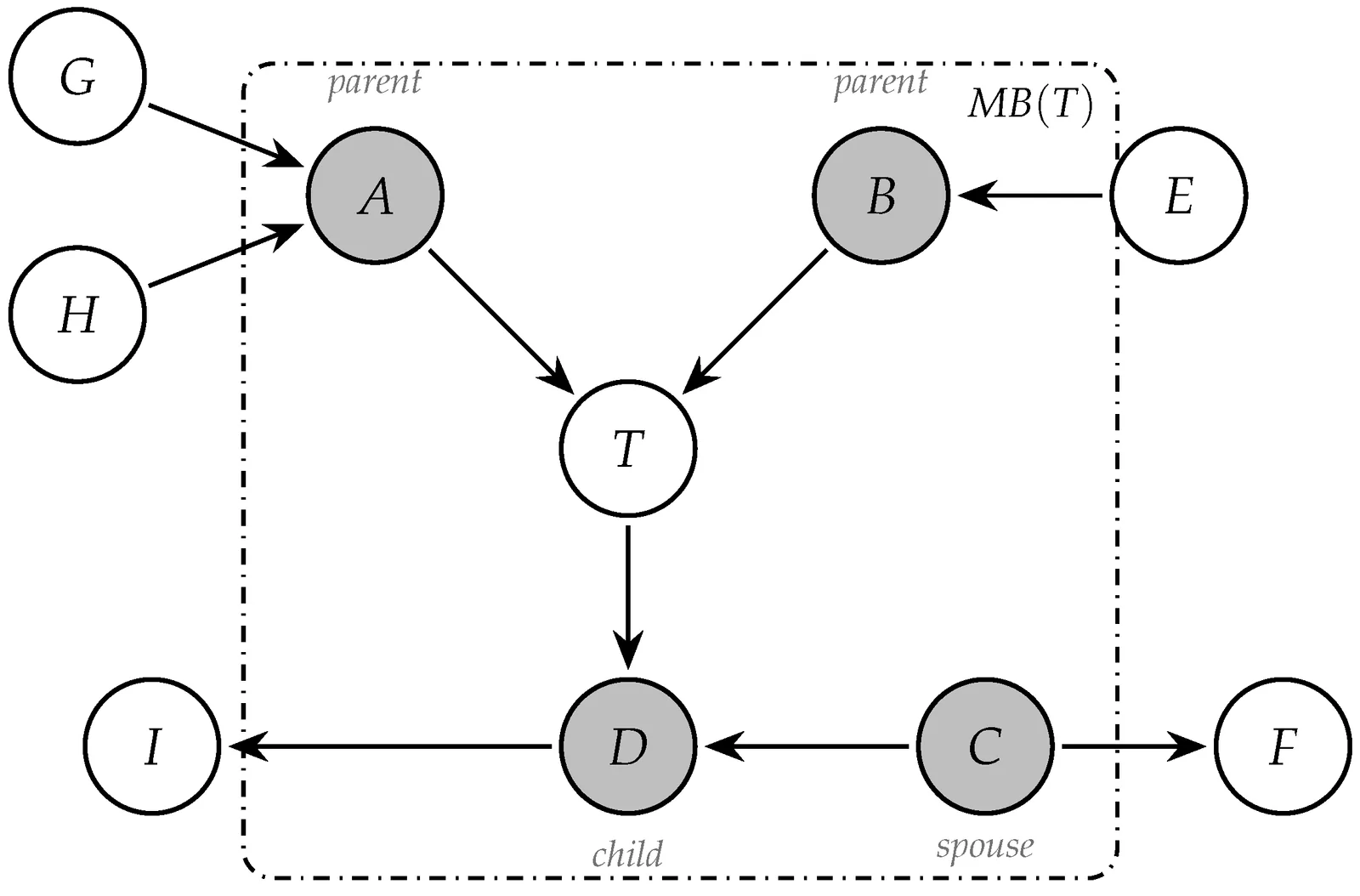
The Markov Blanket of a variable *T* in a Bayesian network (grey nodes). The dash-dot box encloses MB(T)={A,B,C,D}, comprising the parents (A,B), the child (*D*), and the spouse (*C*, the other parent of the child *D*) of *T*.

Like any Bayesian-network-based learner, IPCMB requires the data to be faithful to the underlying BN and the conditional-independence (CI) test to provide controlled error rates. The graphical-model interpretation dates back to Pearl [30]: a directed edge X→Y represents a direct causal influence of *X* on *Y*, and the structure in Figure 2 therefore encodes a causal hypothesis rather than a purely descriptive relationship. Genuine causal recovery rests on three standard conditions: causal sufficiency, faithfulness, and the Markov property. Section 4.1 discusses how the construction of our stock-feature panel makes these conditions plausible.

As outlined in Algorithm 1, IPCMB recovers MB(T) in two stages. The RecognizePC subroutine in Process 1 iteratively prunes any variable from the adjacency set ADJ(T) whose conditional mutual information with *T* (Equation (1)) falls below the threshold ε, growing the conditioning-set size step by step until no further variable can be removed. The surviving set is the candidate parent-and-child set CanPC(T). Process 2 then takes each X∈CanPC(T) and runs RecognizePC again around *X* to detect potential spouses of *T*, applies a symmetry check that drops *X* when *T* does not appear in its own RecognizePC output, and adds any newly identified spouse to the blanket. The final output is MB(T), the union of the parents, children, and spouses of *T*.

Like IAMB [21] and the original PCMB [22], IPCMB belongs to the local-learning family and is well suited to “many variables, few samples” settings. However, its breadth-first design uses fewer conditional-independence tests at each iteration. As documented by Yu et al. [24], the result is a favorable trade-off between the lighter computational footprint of IAMB and the higher selection accuracy of PCMB.

### 3.3. CART

The Classification and Regression Tree (CART) is an efficient non-linear machine-learning model. CART constructs a binary tree in which each node represents a feature and each branch corresponds to a decision rule based on the Gini index shown in Equation (5). The data are recursively partitioned by minimizing the Gini index to maximize node purity, that is, the homogeneity of the observations within each node. This non-linear splitting process allows the decision tree to capture complex relationships among features without imposing a linear functional form.

For a dataset *D* containing |y| classes, its Gini impurity, Gini(D), is defined in Equation (4). Here, $p_{k}$ and $p_{k^{\prime }}$ denote the proportions of observations in *D* that belong to classes *k* and $k^{\prime }$, respectively. Intuitively, Gini(D) reflects the probability that two observations randomly drawn from *D* belong to different classes. A smaller value of Gini(D) therefore indicates greater dataset purity.

(4) $$Gini\left(D\right)=\sum_{k=1}^{\left|y\right|}\sum_{k^{\prime }\neq k}p_{k}p_{k^{\prime }}=1-\sum_{k=1}^{\left|y\right|}p_{k}^{2}$$

The Gini index reaches its minimum value of 0 when all observations belong to a single class. The Gini index associated with feature *a* in dataset *D* is defined as follows:

(5) $$\mathrm{Gini}\;\mathrm{index}\left(D,a\right)=\sum_{v=1}^{V}\frac{|D^{v}|}{\left|D\right|}Gini\left(D^{v}\right)$$

Therefore, among the candidate features in *A*, the feature that produces the lowest post-split Gini index is selected as the optimal splitting feature $a^{*}$.

(6) $$a^{*}=\arg\min_{a\in A}\mathrm{Gini}\;\mathrm{index}\left(D,a\right)$$

Although CART relies on the Gini index, this impurity criterion is closely tied to Shannon entropy and hence to the conditional mutual information used in Section 3.2. The entropy of dataset *D* over |y| classes is

(7) $$H\left(D\right)=-\sum_{k=1}^{\left|y\right|}p_{k}\log_{2}p_{k},$$

and a Taylor expansion of $-\log_{2}p_{k}$ about $p_{k}=1$ shows that $Gini\left(D\right)=1-\sum_{k}p_{k}^{2}$ is a first-order approximation of H(D); both vanish for a pure node and are maximized for a uniform one. Minimizing the Gini index in Equation (5) is therefore approximately equivalent to maximizing the information gain

(8) $$IG\left(D,a\right)=H\left(D\right)-\sum_{v=1}^{V}\frac{|D^{v}|}{\left|D\right|}H\left(D^{v}\right),$$

that is, the reduction in the target’s entropy obtained by partitioning on feature *a*. Consequently, the cumulative impurity reduction that CART attributes to each causal feature—reported as feature importance in Section 4—can be interpreted directly as the information contributed by that feature to the next-month direction forecast. The pipeline therefore rests on a single principle: IPCMB selects the minimal feature set that carries all Shannon information about the target, and CART then partitions that set by greedily maximizing information gain.

CART uses a binary-tree structure and can perform both regression and classification tasks. Unlike black-box models such as neural networks, decision trees offer greater interpretability in stock prediction and can make predictions with a time complexity of $O(\log_{2}n)$, which is generally faster than neural-network inference. However, decision trees are susceptible to overfitting and may settle at local optima, motivating the introduction of causal feature selection in this paper.

## 4. Empirical Research

### 4.1. Data Description and Preprocessing

As an emerging market, the Chinese stock market differs from developed international markets in its trading instruments, accounting standards, and still-developing options and short-selling mechanisms. An analysis of monthly stock returns must therefore account for the institutional characteristics of the Chinese market. Following Wu et al. [10], we select 72 firm characteristics as candidate features. Consistent with standard practice in the machine-learning-based asset-pricing literature [4,5], we classify these features into seven categories: corporate growth, profitability, solvency, valuation, operating efficiency, stock trading, and market and macroeconomic indicators. Appendix A provides the complete list of the 72 candidate features and their abbreviations; detailed definitions follow Wu et al. [10].

Each feature is standardized to zero mean and unit variance before the analysis:

(9) $${\tilde{x}}_{j}=\frac{x_{j}-\mu_{j}}{\sigma_{j}},\;\mu_{j}=\frac{1}{n}\sum_{i=1}^{n}x_{ij},\;\sigma_{j}=\sqrt{\frac{1}{n}\sum_{i=1}^{n}{\left(x_{ij}-\mu_{j}\right)}^{2}},$$

so that each standardized feature ${\tilde{x}}_{j}$ has zero mean and unit variance across the sample. This *z*-score only rescales a feature; it does not by itself make the data normal. The approximate normality that the Fisher-*z* test relies on (Section 3.2) is a separate assumption, and we discuss its role and robustness there. We compute $\mu_{j}$ and $\sigma_{j}$ on the training set alone and reuse them on the test set, so no information from the test period leaks into training.

**Figure 3:**
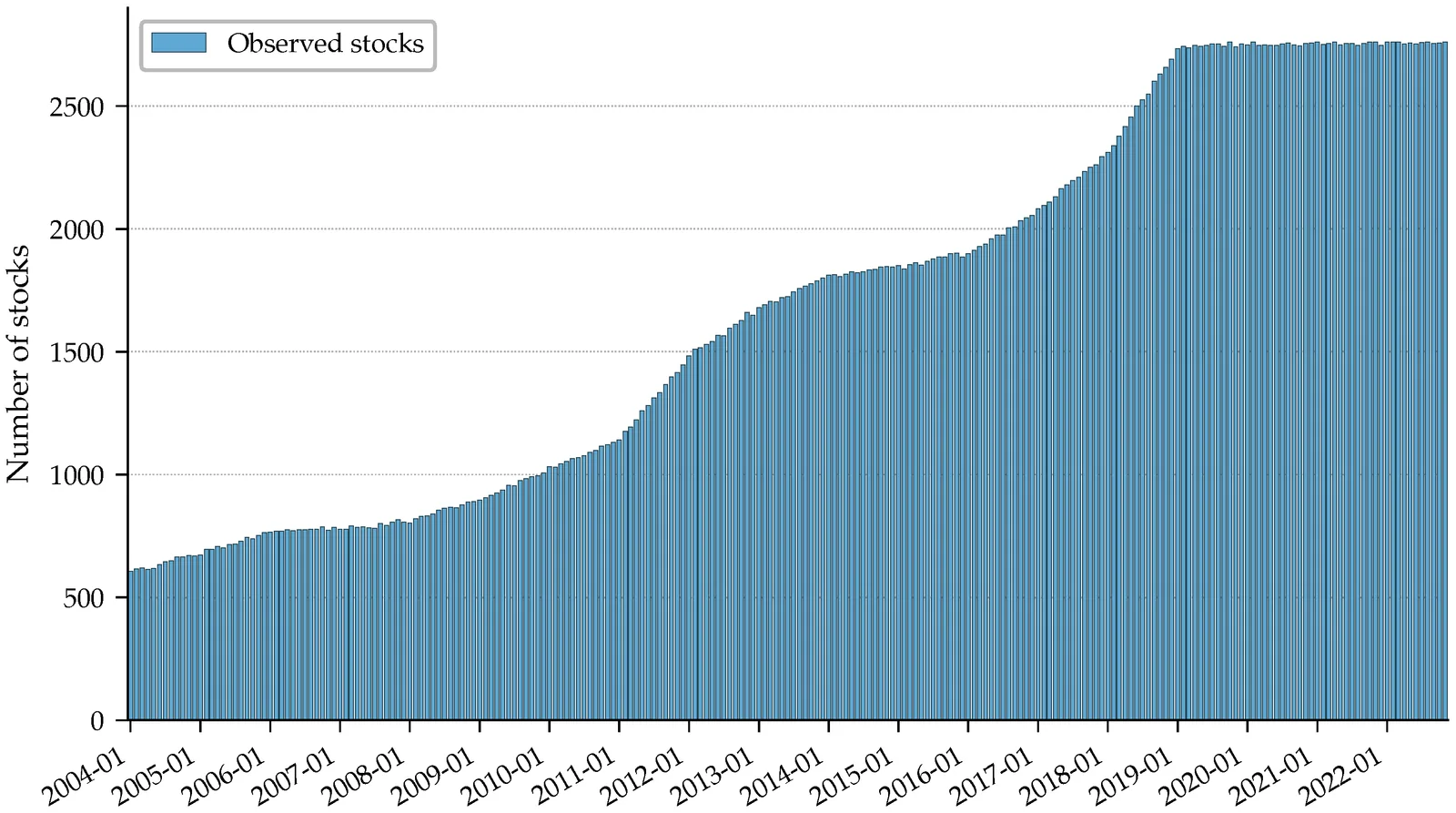
Time distribution of stock data.

The sample covers 2760 stocks from 2004 to 2022. We exclude stocks under special treatment (ST or *ST), stocks listed on the Beijing Stock Exchange, and stocks with short listing histories. After imputing missing data, aligning non-monthly features to the monthly frequency, standardizing all variables according to Equation (9), and converting stock returns into the target indicator, we obtain a dataset of 376,946 observations. All data are sourced from the China Securities Market & Accounting Research Database (CSMAR). We apply the same screening criteria in every period. A stock enters the monthly cross-section if it is an ordinary, non-ST A-share with a complete record and a sufficiently long listing history. Because the filter depends on listing status and data completeness rather than on volatile quantities such as market value or trading volume, the number of eligible stocks changes only gradually. During the test window, it remains close to 2755 per month despite market fluctuations. This stability also reflects the transition to a registration-based listing system, under which the pace of new listings and delistings has been relatively steady. Figure 3 shows the temporal distribution of the stock observations, with the vertical axis indicating the number of observations. It also illustrates the evolving composition of the sample over time. This evolution can limit the effectiveness of traditional feature selection methods and linear prediction models. We therefore apply IPCMB-CART to capture the changing characteristics of the stock data and improve predictive performance.

Before fitting the IPCMB-CART model, the dataset has to satisfy the standard regularity conditions behind BN-based causal discovery. We address this requirement at the data-collection stage by gathering a wide cross-section of monthly stock-return drivers, so that the chance of an unobserved confounder is reduced as far as the available information allows. Three working conditions are then assumed. The causal sufficiency condition requires that the direct causes of monthly stock excess returns are all included in the panel, with no latent variable acting jointly on the target and on the observed predictors. The faithfulness condition requires the empirical dependencies in the data to reflect the dependencies in the true BN, so that the CI test does not mistake genuine signal for noise. The causal Markov condition requires the target to be conditionally independent of its non-descendant nodes given its direct causes, which is what makes prediction from the Markov Blanket sufficient.

None of these conditions holds automatically in financial data. We therefore consider how plausible each is in our setting and what its failure would cost.

Causal sufficiency is the assumption most at risk because some important drivers of returns—most notably investor sentiment—are not observed directly. We mitigate this problem in two ways. First, we collect a broad range of firm-level, trading, and macroeconomic features, leaving less scope for omitted causes. Second, in a market such as China’s, where retail investors dominate, sentiment tends to be reflected in observed variables such as turnover rate (turn_r), short-horizon momentum (mom_3 and mom_6), and the macroeconomic series cpi and gb_5y. If a hidden common cause remains, its effect is likely to be local. The algorithm may miss a spouse node or retain an edge that is not truly causal; the resulting blanket is therefore best interpreted as a strong, compact predictive set rather than a fully identified causal graph.

Faithfulness can fail when two opposing effects cancel and conceal a genuine dependence. In a panel of this size, such exact cancellation is unlikely, although it cannot be ruled out. In either case, the empirical results do not require perfect identification. Most of the twelve Markov Blanket learners compared in Section 5 recover the same core feature set, which outperforms correlation-based feature sets out of sample. This is consistent with the assumptions holding sufficiently well in practice, even if they are never exactly satisfied.

Accordingly, the causal interpretation of the IPCMB results is conditional on these assumptions and should be treated cautiously in observational financial data rather than as definitive causal identification.

### 4.2. Causal Feature Selection

**Figure 4:**
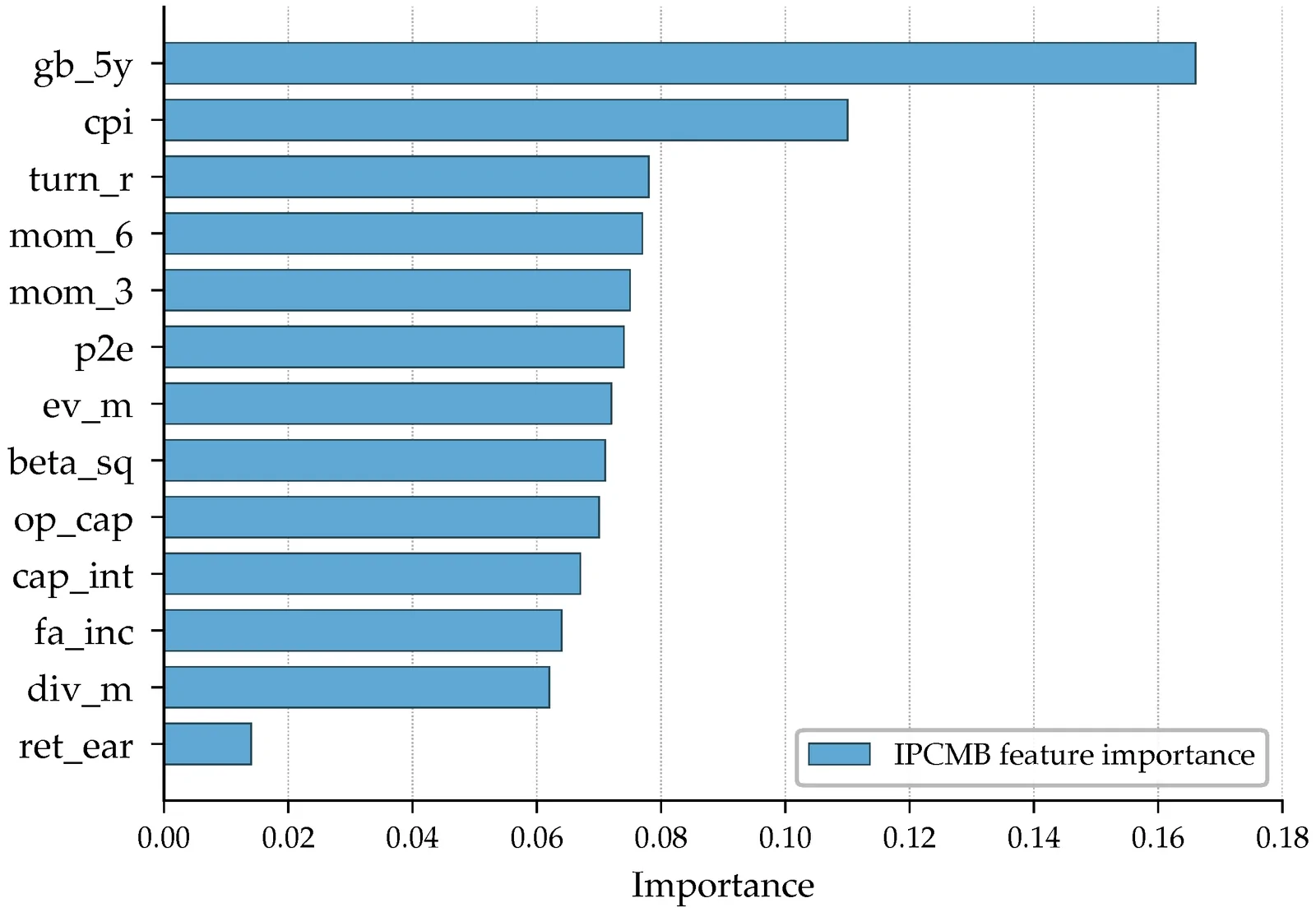
Causal feature importance of the IPCMB-CART model.

When IPCMB is applied to the training set, which covers approximately 90% of the total sample, it returns a Markov Blanket of 13 features that exhibit causal relationships with monthly stock returns in the Chinese market, as shown in Figure 4. The conditional-independence tests use a significance level of α=0.01. A candidate feature is retained only when the Fisher-*z* test of its partial correlation rejects independence at the 1% level, represented by the threshold ε in Algorithm 1. The selected set spans three economic dimensions. At the firm level, IPCMB identifies four valuation and profitability ratios—Price-to-Earnings (p2e), Enterprise Value Multiple (ev_m), Earnings Retention Rate (ret_ear), and Dividend Multiple (div_m)—together with three operating-efficiency proxies: Operating Capital (op_cap), Capital Intensity (cap_int), and Fixed-Assets-to-Revenue Ratio (fa_inc). The trading dimension is represented by Turnover Rate (turn_r), Beta Squared (beta_sq), and three- and six-month momentum (mom_3 and mom_6). At the macroeconomic level, the algorithm retains the 5-year Treasury Bond Return (gb_5y) and the Consumer Price Index (cpi).

Figure 4 displays the causal feature importance computed by the IPCMB-CART model. Because each score is the normalized reduction in impurity (Gini) contributed by a feature across the tree, Equation (8) implies that it approximates the share of information that the causal feature provides about next-month direction. Consistent with Kozak and Santosh [31], bond-related features, particularly Treasury bond returns, exhibit significant causal relationships with monthly stock returns, supporting their reliability as predictors. Consistent with Gu et al. [4], influential predictors of stock returns include price-trend variables (mom_6 and turn_r), valuation variables (ret_ear, p2e, and ev_m), and Beta Squared (beta_sq). However, relative to the findings of Gu et al. [4], the Book-to-Market ratio (btm) may be less effective in the Chinese market than in the U.S. market. One possible explanation is that the explanatory power of the BM factor has declined since the 1970s [32], leading to its exclusion from the proposed model.

More generally, exclusion by IPCMB should be interpreted as conditional redundancy rather than economic irrelevance. For example, ROA and ROE may be redundant once the retained earnings- and valuation-related ratios are known, while 12-month momentum and the 1- and 10-year Treasury bond returns likely overlap with the selected short-horizon momentum and 5-year Treasury bond return, respectively. The omitted predictors therefore add no significant conditional information about next-month direction in this sample.

In addition, the high rankings of the five-year Treasury bond return and cpi indicate their strong influence on Chinese stock returns. One plausible explanation is that movements in China’s stock market are closely tied to government regulation and macroeconomic policy. The Chinese stock market is dominated by retail investors, who tend to exhibit herding behavior in response to policy announcements and buy or sell particular stocks together [5]. This feature distinguishes the Chinese market from the U.S. market and helps explain why gb_5y and cpi are selected as important causal features. For example, on 28 August 2023, the Shanghai Composite Index opened 5.06% higher, mainly in response to the stamp-duty reduction announced the previous day by China’s Ministry of Finance and the China Securities Regulatory Commission.

### 4.3. Rise and Fall Prediction

Following the methodology in Section 3, IPCMB-CART first transforms the target variable, the next month’s stock return, into a binary indicator equal to 0 for a decline and 1 for an increase. The dataset is divided into training and test sets at an approximate ratio of 9:1, yielding 341,126 training observations from January 2004 to October 2021 and 35,820 test observations from November 2021 to November 2022. The split is strictly chronological, with every training month preceding the test months. This design reflects practical forecasting, in which only past data are available when a prediction is made, and avoids the look-ahead bias introduced by a random split. The single split is also demanding because the November 2021–November 2022 test window covers a stressed, declining market. It is not, however, the only test conducted: the rolling backtest in Section 4 re-estimates and reapplies the model month by month, while the four additional historical windows in Section 5 (Table 5) assess stability across markedly different regimes, including the boom-and-bust years of 2008 and 2015. The CART model has a minimum leaf size of 1, a minimum split size of 2, and unrestricted tree depth; it is therefore grown fully without pruning. Consistent with Section 3, causal feature selection rather than a restriction on tree size is used to control overfitting. Implemented in Python 3.9 with Scikit-learn 1.2.2, the model generates a binary forecast of next-month direction from the current month’s causal features, with quarterly and annual variables carried forward uniformly across the corresponding months. To prevent data leakage, causal features are selected using only the training set, ensuring that the IPCMB-CART results remain genuinely out of sample.

We evaluate directional forecasts using standard classification metrics: Accuracy (Acc.), Precision (Pre.), Recall (Rec.), F1 score (F1), and Area Under the ROC (The Receiver Operating Characteristic (ROC) curve is plotted across binary classification thresholds, with the true-positive rate on the y-axis and the false-positive rate on the x-axis). Curve (AUC), as defined in Equations (10)–(14). Table 1 shows the relationships among True Positives (TP), True Negatives (TN), False Positives (FP), and False Negatives (FN). Accuracy measures overall predictive correctness, precision measures the proportion of positive predictions that are correct, and recall, also known as the true-positive rate, measures the proportion of actual positives that are correctly predicted.

(10) $$Accuracy=\frac{TP+TN}{TP+FP+TN+FN}$$

(11) $$Precision=\frac{TP}{TP+FP}$$

(12) $$Recall=\frac{TP}{TP+FN}$$

(13) $$F1=\frac{2\cdot Precision\cdot Recall}{Precision+Recall}=\frac{2TP}{2TP+FP+FN}$$

(14) $$AUC=\frac{\sum_{s_{i}\in P}rank\left(s_{i}\right)-\frac{P\times (P+1)}{2}}{P\times N}$$

**Figure 5:**
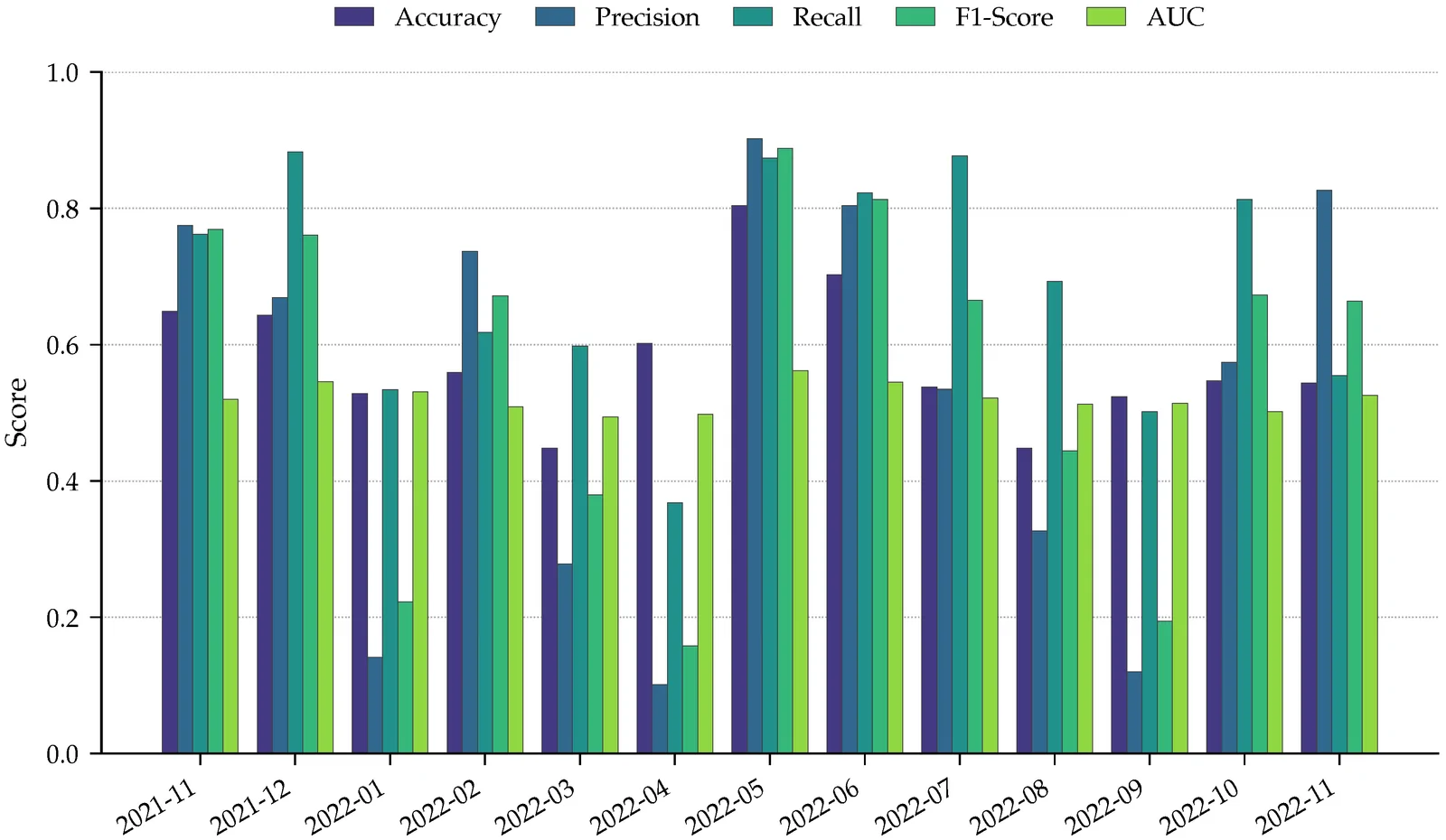
Monthly IPCMB-CART prediction performance.

Finally, the trained IPCMB-CART model uses the selected features to generate rise-or-fall predictions for the test set, which are compared with the realized labels using the metrics defined above. Figure 5 and Table 2 report the monthly results. Across the full 13-month test window, the model achieves an accuracy of 58.0%, precision of 56.9%, recall of 74.3%, an F1 score of 64.5%, and an AUC of 57.6%. These metrics confirm the model’s predictive ability but vary substantially across months. During this period, the Shanghai Composite Index rose in November and December 2021 and in February, May, June, and November 2022, while declining in the remaining months; notably, it fell by 7.65% in January 2022. A comparison of the realized market trends and predictions shows that IPCMB-CART performs better in rising markets, achieving its best result in May 2022 and its worst in March 2022. To indicate class balance in each test month, the “Up” column of Table 2 reports the proportion of stocks with positive returns. The positive-class share varies with the market, ranging from approximately 9% in the sell-off months of April and September 2022 to approximately 92% during the May 2022 rally, with an overall share of 51.6% across the test window. This imbalance largely explains the pronounced monthly variation in the metrics. Precision, in particular, depends on the number of actual rising stocks: it is high in up months, such as May (90.2%) and November 2022 (82.7%), and low in down months, such as April (10.1%). It should therefore be interpreted relative to the monthly base rate rather than in isolation.

These gains also hold up under formal statistical testing. The 58.0% test-set accuracy is well above the 50% random-guess level (binomial test, p<0.001), and a McNemar test on the paired predictions shows that IPCMB-CART is significantly more accurate than the all-features tree ($\chi^{2}=663.4$, p<0.001). For the trading results, the short-only portfolio’s average monthly excess return over the CSI 300 is significantly positive (one-sample *t*-test on the thirteen monthly returns, t=2.70, p=0.02). The model’s edge over both the random baseline and the all-features benchmark is therefore statistically significant. To check whether this predictive ability translates into profitability, the next subsection presents a trading backtest.

**Table 2:** Monthly IPCMB-CART prediction results.

Time	Obs.	Up	Acc.	Pre.	Rec.	F1	AUC
November-2021	2756	76.1%	64.9%	77.5%	76.2%	76.9%	52.0%
December-2021	2756	65.5%	64.3%	66.9%	88.3%	76.1%	54.6%
January-2022	2756	10.2%	52.8%	14.1%	53.4%	22.3%	53.1%
February-2022	2756	76.7%	55.9%	73.7%	61.8%	67.2%	50.9%
March-2022	2756	25.5%	44.8%	27.8%	59.8%	38.0%	49.4%
April-2022	2755	9.8%	60.2%	10.1%	36.8%	15.8%	49.8%
May-2022	2755	91.8%	80.4%	90.2%	87.4%	88.8%	56.2%
June-2022	2755	77.8%	70.3%	80.4%	82.3%	81.3%	54.5%
July-2022	2755	55.8%	53.8%	53.5%	87.7%	66.5%	52.2%
August-2022	2755	32.1%	44.8%	32.7%	69.3%	44.4%	51.3%
September-2022	2755	9.4%	52.4%	12.0%	50.2%	19.4%	51.4%
October-2022	2755	57.9%	54.7%	57.4%	81.3%	67.3%	50.2%
November-2022	2755	82.5%	54.4%	82.7%	55.5%	66.4%	52.6%
Total	35,820	51.6%	58.0%	56.9%	74.3%	64.5%	57.6%

### 4.4. Backtest of the IPCMB-CART Model

**Figure 6:**
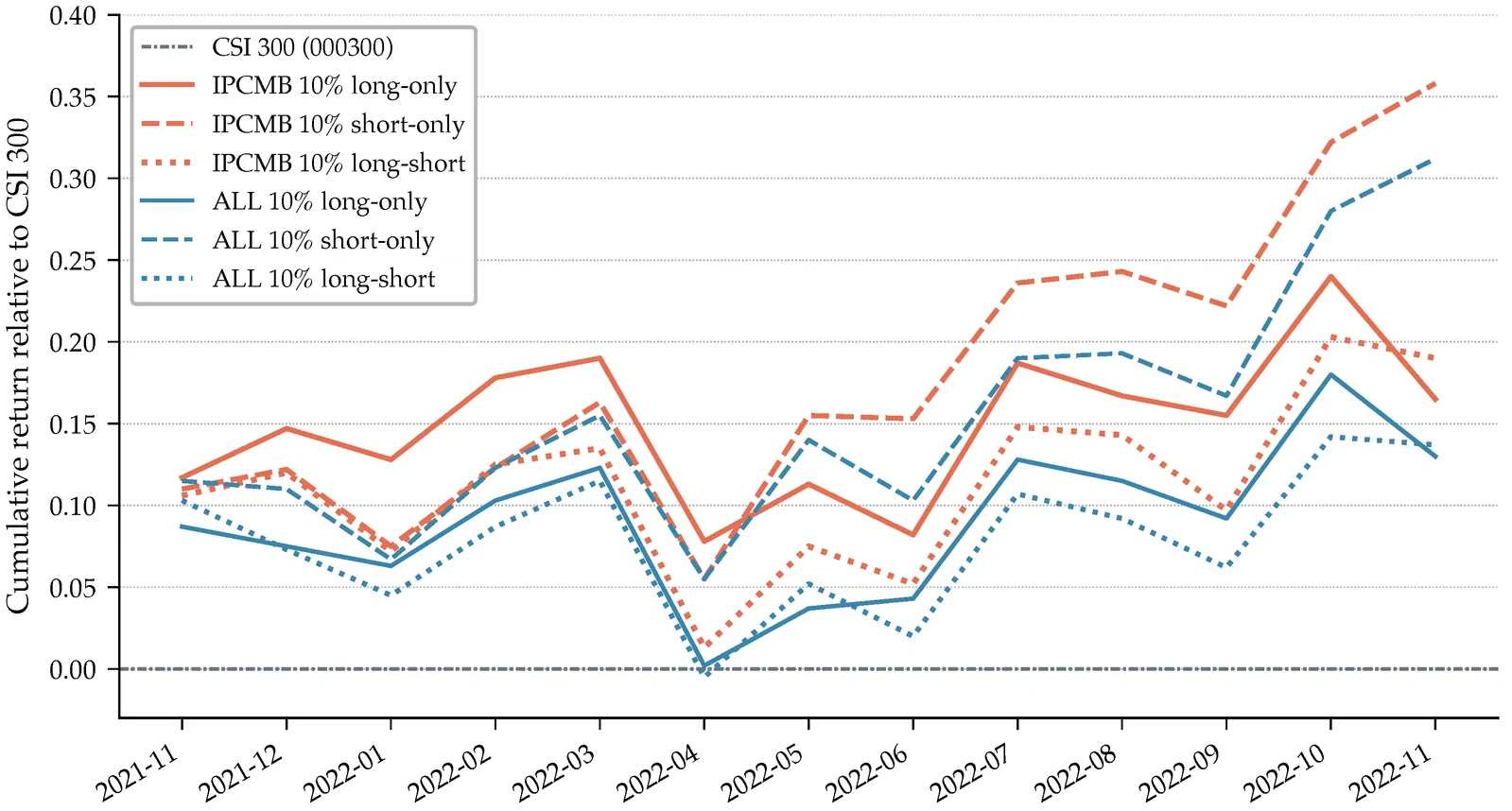
Backtest line chart of cumulative stock returns over 13 months.

To assess whether IPCMB-CART can generate cumulative investment returns, we conduct rolling forecasts and backtests on the test set using historical data. Although the Chinese stock market lacks a fully developed short-selling mechanism, the analysis assumes that stocks can be shorted effectively. We backtest three strategies using the trained IPCMB-CART model: long-only, short-only, and long-short. The long-only strategy selects the top 10% of stocks by current-month return from among those predicted to rise in the following month, forms an equally weighted portfolio, and holds it for one month before rebalancing; this process is repeated over the 13-month test period. Conversely, the short-only strategy selects the bottom 10% of stocks with the most negative predicted returns. The long-short strategy simultaneously buys the top 5% and shorts the bottom 5% of stocks. The CSI 300 Index (China Securities Index 300, code: 0003000) serves as the benchmark for calculating relative cumulative returns. Figure 6 and Table 3 present the results.

Figure 6 shows the 13-month cumulative returns of the long-only (solid line), short-only (dashed line), and long-short (dotted line) strategies based on IPCMB-CART (red) and CART trained on all features (black). Because the Chinese stock market declined persistently from November 2021 to November 2022 and the Shanghai Composite Index fell by 10.3% over the period, the short-only strategies generated substantial cumulative returns. Among the six strategies, shorting the bottom 10% of stocks selected by IPCMB-CART generated the highest cumulative return, outperforming the CSI 300 Index by 35.88% over 13 months. A comparison of the red and black lines shows that IPCMB-CART outperforms the all-features decision tree across the different portfolios, supporting the effectiveness of the proposed model.

**Table 3:** Backtest results for cumulative stock returns over 13 months.

Portfolio	Model	Average Return	Cumulative Return	Sharpe Ratio
10% short-only	IPCMB-CART	2.99%	35.88%	13.47
	ALL-CART	2.61%	31.32%	14.41
10% long-only	IPCMB-CART	1.38%	16.58%	12.54
	ALL-CART	1.08%	12.96%	9.45
10% long-short	IPCMB-CART	1.57%	18.80%	12.27
	ALL-CART	1.13%	13.57%	10.73

Note: Stock returns and Sharpe ratios are calculated monthly relative to the return of the CSI 300 Index.

The CSI 300 Index is treated as an investable portfolio. Equation (15) is used to calculate the Sharpe ratios reported in Table 3, where $R_{p}$ denotes the expected return of portfolio *p*, $R_{f}$ is the risk-free rate, and $\sigma_{p}$ is the standard deviation of the portfolio return. The Sharpe ratios in Table 3 are large, and their construction is important for interpretation. They are calculated at the monthly frequency and are not annualized. Specifically, $E(R_{p})$ and $\sigma_{p}$ are the mean and standard deviation of the 13 monthly portfolio returns measured in excess of the CSI 300. With only 13 observations and a strongly trending market, $\sigma_{p}$ is small, mechanically increasing the ratio. We therefore interpret these values as within-sample comparisons across portfolios rather than as the annualized Sharpe ratios of live strategies; their relative ordering is more informative than their absolute levels.

IPCMB-CART generates higher returns than the decision-tree portfolio trained on all features (ALL-CART). As shown in Figure 6, the returns from shorting stocks selected by IPCMB-CART and ALL-CART were nearly identical before May 2022. However, as IPCMB-CART’s predictive accuracy increased markedly in May and June (Figure 5), the return gap between the two models widened substantially.

The backtest is deliberately simple. It assumes frictionless short selling and monthly rebalancing with equal weights, while ignoring transaction costs, slippage, and liquidity. Because the portfolio turns over once per month, a typical round-trip cost for Chinese A-shares—approximately 0.1–0.3% after accounting for commissions and stamp duty—would reduce each month’s return by roughly the same amount. Relative to an average monthly gain of 2.99% and a cumulative edge of 35.88% over the benchmark, the short-only strategy would remain clearly profitable after such costs. A more comprehensive model of execution costs and borrowing fees would refine these estimates. However, because this study predicts only the direction, rather than the magnitude, of next-month stock returns, the simple strategies and portfolios based on current-month returns are not directly suitable for practical investment. Further robustness tests of the IPCMB-CART results are therefore warranted.

(15) $$Sharpe\;Ratio=\frac{E\left(R_{p}\right)-R_{f}}{\sigma_{p}}$$

## 5. Robustness Tests

### 5.1. Robustness Tests of Data Samples

#### 5.1.1. Winsorization

Newly listed stocks often exhibit substantial volatility, particularly on the first trading day or during the first month. For instance, in the first quarter of 2024, the average first-day gain of newly listed stocks on the Shanghai and Shenzhen exchanges was approximately 99%, about 30% higher than in 2023. Such volatility arises because the market is uncertain about the valuation of a newly listed firm and investors hold divergent expectations about its prospects. In addition, Chinese stock prices often fluctuate sharply when a firm is about to receive an ST (Special Treatment) designation or be delisted, prompting retail investors to liquidate their positions quickly. To mitigate the influence of extreme values and improve predictive reliability and stability, we winsorize the dataset at the 1%, 5%, and 10% levels. Winsorization limits extreme observations to specified percentiles of the data. Table 4 reports the IPCMB-CART direction-prediction results obtained from these processed datasets.

As the degree of winsorization increases, IPCMB-CART’s performance metrics decline. Excessive winsorization may remove large stock-price movements that contain information about the market’s primary investment direction. Although an appropriate degree of winsorization can generally improve predictive performance, IPCMB-CART achieves its highest accuracy of 58.0% without winsorization (Table 4). This result suggests that the model can already limit the influence of extreme values while accurately identifying causal features, so winsorization has little additional effect. Winsorization likewise fails to improve the predictive accuracy of ALL-CART, possibly because noisy features continue to influence its predictions. Filtering out such irrelevant features is therefore essential to improving the predictive performance of machine-learning models.

#### 5.1.2. Different Periods

The COVID-19 pandemic adversely affected the Chinese market, and stock prices followed an overall downward trend in 2022. To reduce the influence of this exceptional event and assess the robustness of IPCMB-CART, we test the model over four additional periods: (1) 2004–2019, before the COVID-19 pandemic, with the 13 months of 2019 used as the test set; (2) 2004–2015, which includes a period of stock-market turbulence; (3) 2004–2008, which includes the financial crisis; and (4) 2004–2013, which exhibits a relatively stable but oscillating trend. The Chinese stock market rose and then fell rapidly in both 2008 and 2015, making these years markedly different from more stable periods. Effective prediction of market direction across these periods would therefore support the robustness of IPCMB-CART.

Table 5 shows that IPCMB-CART performs strongly across periods, attaining its highest accuracy of 61.4% for the 2004–2019 sample. Excluding the COVID-19 years likely removes a major macroeconomic shock from the feature space, allowing the causal features to provide clearer signals. The result also suggests that macroeconomic variables, particularly those that capture abrupt events, are important determinants of both stock trends and model accuracy. By contrast, ALL-CART performs noticeably worse in every subperiod, achieving only 45.0% accuracy for 2004–2008, when the shorter data window also constrains learning. The weaker results for the volatile 2008 and 2015 windows relative to the more stable 2013 and 2019 windows further indicate that market regimes affect the predictive ability of tree-based models. Taken together, these four windows provide a simple multiperiod stability check. Each uses a different chronological split, and the test years cover markedly different regimes: the 2008 financial crisis, the 2015 boom and bust, the calmer market of 2013, and the pre-pandemic market of 2019. IPCMB-CART outperforms ALL-CART in every case, although its accuracy understandably declines in the most turbulent years. Thus, the model’s advantage is not confined to a single regime or the COVID-19 period, and this evidence complements the month-by-month rolling backtest in Section 4.

### 5.2. Comparison of CFS Algorithms

**Figure 7:**
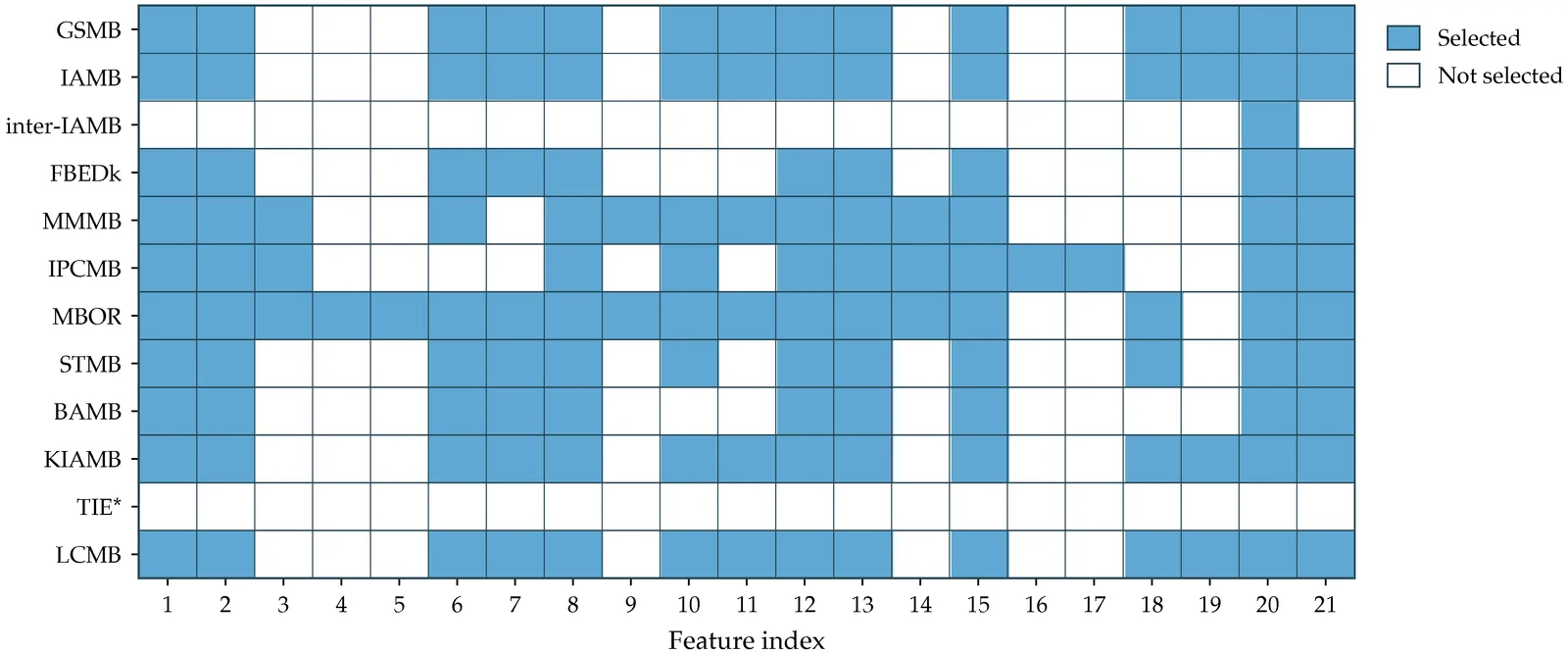
Selection results of different CFS algorithms.

Building on the preceding theory and methods, we compare 12 causal feature selection (CFS) algorithms: four simultaneous MB learning methods (GSMB, IAMB, inter-IAMB, and FBEDk), four stepwise MB learning methods (MMMB, IPCMB, MBOR, and STMB), one hybrid MB learning method (BAMB), and three MB learning methods based on relaxed assumptions (KIAMB, TIE*, and LCMB). Using the pyCausalFS module [24], each algorithm selects causality-based features from the 72 candidates associated with monthly stock returns [10]. Figure 7 reports the results after omitting features that were not selected by any of the 12 algorithms. The vertical axis lists the CFS algorithms, the horizontal axis lists the monthly stock-return features, gray cells indicate selected features, and white cells indicate unselected features.

Most CFS algorithms select the following eight features: Dividend Multiple (div_m), Earnings Retention Rate (ret_ear), Enterprise Value Multiple (ev_m), Fixed Assets to Income Ratio (fa_inc), Capital Intensity (cap_int), Beta Squared (beta_sq), Five-Year Treasury Bond Return (gb_5y), and Consumer Price Index (cpi). The IAMB-based algorithms—GSMB, IAMB, FBEDk, KIAMB, and LCMB—produce consistent feature sets. GSMB, IAMB, and FBEDk are simultaneous MB learning algorithms, whereas KIAMB and LCMB are derivatives of IAMB. By contrast, inter-IAMB identifies gb_5y as the sole decisive factor for monthly stock returns, while TIE* fails to select any effective features. The agreement across algorithms provides evidence of stability. Because the twelve learners differ in their search order, conditioning sets, and independence tests, a feature recovered by most of them is unlikely to be an artifact of a single procedure. In this sense, the eight characteristics listed above, which form the core of the IPCMB blanket, are robust; features selected by only one algorithm should be treated as sample- or method-specific. Reapplying IPCMB to bootstrap resamples of the training set and recording each feature’s selection frequency would measure this stability more directly and provide a useful complement to the present results.

Table 6 shows that the feature subsets selected by GSMB, IAMB, KIAMB, and LCMB perform poorly in stock-direction prediction, whereas the remaining seven CFS algorithms outperform the 50.3% accuracy achieved using all 72 features. IPCMB provides the best overall predictive performance, with an accuracy of 58.0%, precision of 56.9%, an F1 score of 64.5%, and an AUC of 57.6%. STMB achieves the highest recall, at 75.2%. Although divide-and-conquer MB learning methods such as MMMB, IPCMB, MBOR, and STMB are less computationally efficient, their selected feature subsets predict stock-price direction more effectively than those produced by the other three categories of MB learning methods.

The poor predictive performance of full-feature learning may result from the excessive number of predictors, as noise and redundant information reduce the model’s ability to generalize. Some features may have little relationship with monthly returns or no significant causal association and therefore act as noise variables. The model may be unable to identify and exclude these irrelevant features, causing it to focus on noise and idiosyncratic details in the training data while overlooking broader patterns. By contrast, the features selected by CFS algorithms are more representative, retain key information related to monthly Chinese stock returns, and reduce the risk of overfitting.

### 5.3. Comparison of Feature Selection Algorithms

**Figure 8:**
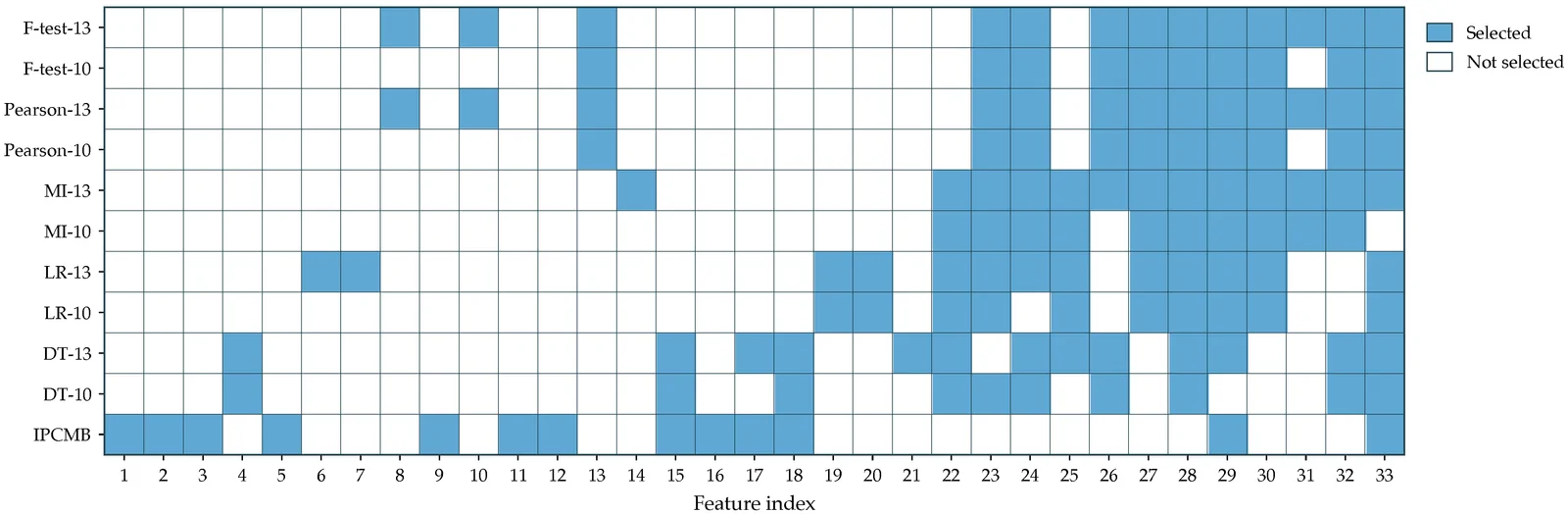
Selection results of different feature selection algorithms.

To further assess the feature-selection performance of CFS algorithms, we compare IPCMB with the filter methods based on the F-test, Pearson correlation coefficient, and Mutual Information (MI), as well as the embedded methods based on Logistic Regression (LR) and Decision Tree (DT). Because IPCMB selects 13 causal features, we take the 13 highest-ranked features from each competing algorithm. For example, after an LR model is trained, each of the 72 features receives a weight, and the 13 features with the largest weights form the embedded feature-selection result. We also evaluate these methods using their 10 highest-ranked features because, unlike IPCMB, they cannot adaptively determine the number of effective features. Figure 8 shows the selection results for the different algorithms.

Compared with the CFS results in Figure 7, traditional feature selection algorithms are more likely to select market-trading variables, such as A-share Dividend Yield (a_dy), A-share Turnover Rate (a_turn), and the Risk-Free Rate (rf), than firm fundamentals. One possible explanation is that, from a correlational perspective, most stocks co-move closely with the broad market index. However, because the index reflects the aggregate performance of its weighted constituent stocks, many studies [4,5] do not use index variables to predict next-month stock direction. Some CFS algorithms also select the A-share Book-to-Market Ratio (a_btm), as shown in Figure 7. Moreover, both CFS and traditional feature selection identify macroeconomic factors—particularly the 5-Year Treasury Bond Return (gb_5y) and Consumer Price Index (cpi)—as effective predictors, supporting their role as major causal features of stock-return direction. Specifically, traditional feature selection also identifies the 1-, 5-, and 10-year Treasury Bond Returns (gb_1y, gb_5y, and gb_10y) as important determinants of monthly stock returns.

Table 7 reports the predictive performance of the different feature selection algorithms. IPCMB, with 13 selected features, achieves the highest accuracy, recall, F1 score, and AUC, whereas MI with 10 features yields the lowest accuracy (42.7%) and AUC (42.4%). The widely used Pearson correlation coefficient also produces relatively weak predictions, with an accuracy of only approximately 52%. Embedded feature selection based on LR and DT appears more reliable. However, because machine-learning optimization can involve stochastic variation, the selected features may not yield equally stable results in every run. Overall, the findings indicate that CFS algorithms have considerable potential for stock prediction. The experiments also show the extent to which performance depends on the number of selected features, which IPCMB determines adaptively rather than manually. Evaluating the competing methods with 10 and 13 features brackets the size of the IPCMB blanket, yet the 13 features chosen by IPCMB outperform every fixed-size subset. Thus, the identity of the retained features matters more than their number. Selecting fewer features than the blanket omits predictors that still contain information about the target, whereas moving toward the full set—as in the ALL-feature result of 50.3%—reintroduces the redundant and noisy predictors that the blanket is designed to exclude.

This gap has a natural information-theoretic explanation. Ranking methods such as Mutual Information score each feature by its marginal information about the target, $I(X_{i};T)$, and keep the top-ranked ones. Such scoring is blind to redundancy (two highly informative features may carry almost the same information about *T*) and to synergy (a feature that is weak on its own may become decisive once combined with others). IPCMB instead evaluates each feature by its conditional information given the others, $I(X_{i};T|S)$ (Equation (1)), and returns the Markov Blanket, the minimal set beyond which the remaining features supply no further information about *T* (Equation (3)). It thus discards features that are merely correlated proxies of stronger predictors while retaining synergistic ones, which is why the IPCMB subset outperforms the marginal-MI and correlation-based subsets of comparable size in Table 7.

### 5.4. Comparison of Prediction Models

We also compare the performance of prediction models trained on either all features or the subset selected by IPCMB. The models include Naive Bayes (NB), K-Nearest Neighbors (KNN), Logistic Regression (LR), Classification and Regression Tree (CART), and a simple single-layer Artificial Neural Network (ANN). The neural network is configured with 50 hidden nodes, trained for 10 epochs, and optimized using the Adam optimizer implemented in PyTorch 1.13.1. Table 8 reports the results. IPCMB-CART performs best, whereas ALL-ANN performs worst, with an accuracy of 45.6%.

All prediction models perform better when trained on the IPCMB feature subset than when trained on all features. In particular, IPCMB-CART and IPCMB-ANN achieve substantially higher accuracy than their all-feature counterparts. However, ALL-LR records the highest recall, at 94.5%. This high recall may reflect bias induced by features such as rf, causing ALL-LR to predict predominantly positive price movements. In terms of computational time, using all features increases the time required by some models, including NB and LR, whereas ALL-KNN requires considerably less time than IPCMB-KNN.

## 6. Conclusions

This paper presents IPCMB-CART, an information-theoretic, causality-based model for stock prediction. The model recovers the Markov Blanket of next-month returns through conditional mutual information tests and passes the selected features to a tree whose splits maximize information gain. In doing so, it addresses the causality, interpretability, and high-dimensionality challenges faced by traditional algorithms in non-linear, evolving stock markets. Backtesting and robustness tests support the model’s effectiveness and generalizability. It achieves a predictive accuracy of 58.0% and a 13-month cumulative return 35.88% above that of the CSI 300 Index. Compared with traditional feature selection methods, including the Pearson correlation coefficient, Mutual Information, and embedded LR, as well as prediction models such as Naive Bayes and KNN, IPCMB-CART achieves the highest accuracy and AUC.

In practical terms, the results suggest that introducing causal feature selection during preprocessing can give investors a small but consistent edge in the Chinese A-share market while keeping the prediction model sufficiently interpretable for inspection by portfolio managers and regulators. This consideration is particularly relevant to emerging markets, where retail investors and policy shocks make returns more difficult to forecast using purely correlation-based factors. Two limitations should be acknowledged. First, because of data-availability constraints, only Chinese-market data are used for robustness testing, and the model’s external validity in other markets remains to be established. Second, the model predicts only the direction of next-month returns, not their magnitude, which limits its direct application to practical portfolio construction. The simplifications that make the present study manageable also point to clear directions for future research. These include replacing the Gaussian test with a nonparametric conditional mutual information or copula-based test, measuring Markov Blanket stability through bootstrap resampling, comparing the unpruned tree with pruned and depth-limited alternatives, and incorporating realistic trading costs and short-borrow fees into the backtest. Future studies could also combine additional CFS algorithms, extend the analysis to other emerging markets, and integrate return-magnitude forecasts with richer information-theoretic measures, such as transfer entropy, to support risk-aware trading strategies.

## Figures and Tables

**Table 1:** Relationships between actual and predicted classes.

		Real	
		Positive	Negative
**Predicted**	Positive	TP	FP
	Negative	FN	TN

Note: TP (True Positives) denotes observations correctly predicted as positive; TN (True Negatives) denotes observations correctly predicted as negative; FP (False Positives) denotes observations incorrectly predicted as positive; and FN (False Negatives) denotes observations incorrectly predicted as negative.

**Table 4:** Robustness results after winsorization.

Model	Winsorization	Acc.	Pre.	Rec.	F1	AUC
ALL-CART	no	50.3%	50.7%	46.4%	48.4%	50.4%
	1%	49.5%	49.5%	45.6%	47.5%	49.5%
	5%	50.1%	50.6%	39.3%	44.2%	50.2%
	10%	48.3%	49.4%	42.4%	45.6%	48.5%
IPCMB-CART	no	58.0%	56.9%	74.3%	64.5%	57.6%
	1%	57.8%	56.5%	67.9%	61.7%	57.8%
	5%	57.1%	56.0%	68.7%	61.7%	57.0%
	10%	55.9%	55.7%	66.9%	60.8%	55.6%

**Table 5:** Robustness results across different periods.

Model	Periods	Acc.	Pre.	Rec.	F1	AUC
ALL-CART	2004–2022	50.3%	50.7%	46.4%	48.4%	50.4%
	2004–2019	53.2%	49.7%	49.3%	49.5%	52.9%
	2004–2015	46.9%	53.0%	60.3%	56.4%	44.7%
	2004–2013	48.8%	52.1%	41.2%	46.0%	49.3%
	2004–2008	45.0%	41.1%	60.0%	48.8%	46.7%
IPCMB-CART	2004–2022	58.0%	56.9%	74.3%	64.5%	57.6%
	2004–2019	61.4%	66.0%	66.9%	66.4%	60.6%
	2004–2015	54.7%	59.8%	62.9%	61.3%	53.4%
	2004–2013	57.1%	61.0%	64.3%	62.6%	56.2%
	2004–2008	57.0%	51.0%	38.7%	44.0%	55.0%

**Table 6:** Prediction results for different CFS algorithms.

Method	Algorithms	N	Acc.	Pre.	Rec.	F1	AUC
ALL-feature learning	—	72	50.3%	50.7%	46.4%	48.4%	50.4%
Simultaneous MB learning	GSMB	14	48.2%	48.6%	54.9%	51.6%	48.1%
	IAMB	14	48.5%	49.0%	58.7%	53.4%	48.4%
	inter-IAMB	1	53.9%	53.1%	69.5%	60.2%	53.8%
	FBEDk	10	54.7%	53.7%	72.1%	61.5%	54.6%
Divide-and-conquer MB learning	MMMB	14	56.0%	54.6%	74.1%	62.9%	55.9%
	IPCMB	13	58.0%	56.9%	74.3%	64.5%	57.6%
	MBOR	18	56.0%	54.5%	75.1%	63.2%	55.9%
	STMB	12	56.2%	54.7%	75.2%	63.3%	56.1%
Interleaving MB learning	BAMB	10	53.1%	52.6%	66.6%	58.8%	53.0%
Assumption-relaxed MB learning	KIAMB	14	49.3%	49.7%	59.9%	54.3%	49.2%
	TIE*	0	—	—	—	—	—
	LCMB	14	47.5%	48.0%	54.1%	50.9%	47.5%

**Table 7:** Prediction results for different feature selection algorithms.

Algorithms	N	Acc.	Pre.	Rec.	F1	AUC
ALL-feature	72	50.3%	50.7%	46.4%	48.4%	50.4%
IPCMB	13	58.0%	56.9%	74.3%	64.5%	57.6%
F-test-13	13	53.1%	56.3%	38.0%	45.4%	53.5%
F-test-10	10	52.8%	55.6%	40.0%	46.5%	53.2%
Pearson-13	13	52.0%	54.6%	37.8%	44.7%	52.4%
Pearson-10	10	52.1%	53.9%	45.1%	49.1%	52.3%
MI-13	13	54.4%	56.4%	48.1%	51.9%	54.5%
MI-10	10	42.7%	45.1%	54.2%	49.2%	42.4%
LR-13	13	50.5%	51.8%	51.5%	51.6%	50.5%
LR-10	10	55.7%	56.1%	62.3%	59.0%	55.5%
DT-13	13	56.3%	58.5%	51.1%	54.6%	56.5%
DT-10	10	48.0%	49.0%	38.7%	43.3%	48.2%

**Table 8:** Prediction results for different prediction models.

Feature Set	Model	Acc.	Pre.	Rec.	F1	AUC	Time
ALL-feature	NB	49.8%	50.9%	55.8%	53.3%	49.6%	0.3
	KNN	47.8%	49.1%	47.1%	48.1%	47.8%	14.0
	LR	51.5%	51.5%	94.5%	66.6%	50.4%	7.3
	CART	50.3%	50.7%	46.4%	48.4%	50.4%	42.6
	ANN	45.6%	46.1%	36.3%	40.6%	45.8%	127.7
IPCMB	NB	51.3%	51.5%	89.2%	65.3%	50.3%	0.1
	KNN	51.5%	52.4%	59.0%	55.5%	51.3%	72.4
	LR	54.3%	54.2%	70.2%	61.2%	53.9%	7.2
	CART	58.0%	56.9%	74.3%	64.5%	57.6%	8.0
	ANN	53.7%	53.0%	85.3%	65.4%	52.9%	132.3

## Data Availability

The data used in this study were obtained from the China Stock Market & Accounting Research (CSMAR) database. Restrictions apply to the redistribution of these data; they are available from the authors upon reasonable request and with the permission of CSMAR.

## Abbreviations

The following abbreviations are used in this manuscript:

ANN	Artificial Neural Network
ARMA	Autoregressive Moving Average
AUC	Area Under the ROC Curve
BN	Bayesian Network
CART	Classification and Regression Tree
CFS	Causal Feature Selection
CI test	Conditional Independence Test
CMI	Conditional Mutual Information
CPI	Consumer Price Index
CSI	China Securities Index
CSMAR	China Securities Market & Accounting Research Database
DT	Decision Tree
IG	Information Gain
IPCMB	Iterative Parent–Child-based search of Markov Blanket
KNN	K-Nearest Neighbors
LR	Logistic Regression
LSTM	Long Short-Term Memory
MB	Markov Blanket
MI	Mutual Information
NB	Naive Bayes
PCA	Principal Component Analysis
PCC	Pearson Correlation Coefficient
ROC	Receiver Operating Characteristic
SVM	Support Vector Machine
TBR	Treasury Bond Return

## Appendix A. Full List of Candidate Features

For reproducibility, Table A1 lists all 72 candidate features entered into the feature-selection stage, together with their abbreviations. Their construction and detailed definitions follow Wu et al. [10].

**Table A1:** List of the 72 candidate features.

No.	Features	Abb.	No.	Features	Abb.
1	Fixed Asset Growth Rate	fa_g	37	Asset–liability Ratio	dar
2	Total Asset Growth Rate	ta_g	38	Equity Multiplier	em
3	Net Profit Growth Rate	np_g	39	Equity to Debt Ratio	e2d
4	Total Profit Growth Rate	tp_g	40	Accounts Receivable Turnover Ratio	ar_turn
5	Operating Profit Growth Rate	op_g	41	Inventory to Revenue Ratio	inv_r
6	Revenue Growth Rate	rev_g	42	Inventory Turnover Ratio	inv_turn
7	Sustainable Growth Rate	sus_g	43	Accounts Payable Turnover Ratio	ap_turn
8	Owner’s Equity Growth Rate	oe_g	44	Operating Capital Turnover Ratio	opc_turn
9	Net Asset Growth per Share	na_g	45	Cash and Cash Equivalent Turnover Ratio	cash_turn
10	After-Tax Cash Dividends per Share	div_at	46	Current Assets to Income Ratio	ca_inc
11	Dividend Multiple	div_m	47	Current Asset Turnover Ratio	ca_turn
12	Earnings Retention Ratio	ret_ear	48	Fixed Assets to Income Ratio	fa_inc
13	Price–Earnings Ratio	p2e	49	Fixed Asset Turnover Rate	fa_turn
14	Price–Sales Ratio	p2s	50	Non-Current Asset Turnover Rate	nca_turn
15	Price–Book Ratio	p2b	51	Capital Intensity	cap_int
16	Market Value to Tangible Assets Ratio	mv_ta	52	Total Asset Turnover Rate	ta_turn
17	Market Value	mv	53	Beta	beta
18	Tobin’s Q Ratio	tobin_q	54	Beta Squared	beta_sq
19	Book-to-Market Ratio	btm	55	Circulating Shares Monthly Turnover Rate	turn_r
20	Enterprise Value Multiple	ev_m	56	3-Month Momentum	mom_3
21	Return on Assets	roa	57	6-Month Momentum	mom_6
22	Return on Total Assets	rota	58	12-Month Momentum	mom_12
23	Return on Equity	roe	59	Long-Term Reversal	lt_rev
24	Earnings Before Interest and Taxes	ebit	60	Short-Term Reversal	st_rev
25	Return on Invested Capital	roic	61	A-share Price–Earnings Ratio	a_pe
26	Long-Term Return on Capital	ltrc	62	A-share Dividend Yield	a_dy
27	Gross Operating Margin	gom	63	A-share Turnover Rate	a_turn
28	Net Operating Margin	nom	64	A-share Volatility	a_vol
29	Operating Margin Before Interest and Taxes	ombit	65	A-share Book-to-Market Ratio	a_btm
30	Income Tax Rate	itax	66	Risk-Free Rate	rf
31	Comprehensive Tax Rate	ctax	67	1-Year Treasury Bond Return	gb_1y
32	Current Ratio	cur_r	68	5-Year Treasury Bond Return	gb_5y
33	Quick Ratio	quick_r	69	10-Year Treasury Bond Return	gb_10y
34	Cash Ratio	cash_r	70	Trade Surplus	ts
35	Operating Capital	op_cap	71	Default Spreads	ds
36	Interest Coverage Ratio	int_cov	72	Consumer Price Index	cpi
